# Inorganic Nanoparticle Functionalization Strategies in Immunotherapeutic Applications

**DOI:** 10.34133/bmr.0086

**Published:** 2024-09-25

**Authors:** Wei Mao, Hyuk Sang Yoo

**Affiliations:** ^1^ Department of Biomedical Materials Engineering, Kangwon National University, Chuncheon 24341, Republic of Korea.; ^2^ Institute for Molecular Science and Fusion Technology, Kangwon National University, Chuncheon 24341, Republic of Korea.; ^3^ Institute of Biomedical Science, Kangwon National University, Chuncheon 24341, Republic of Korea.; ^4^ Kangwon Radiation Convergence Research Center, Kangwon National University, Chuncheon 24341, Republic of Korea.

## Abstract

Nanotechnology has been increasingly utilized in anticancer treatment owing to its ability of engineering functional nanocarriers that enhance therapeutic effectiveness while minimizing adverse effects. Inorganic nanoparticles (INPs) are prevalent nanocarriers to be customized for a wide range of anticancer applications, including theranostics, imaging, targeted drug delivery, and therapeutics, because they are advantageous for their superior biocompatibility, unique optical properties, and capacity of being modified via versatile surface functionalization strategies. In the past decades, the high adaptation of INPs in this emerging immunotherapeutic field makes them good carrier options for tumor immunotherapy and combination immunotherapy. Tumor immunotherapy requires targeted delivery of immunomodulating therapeutics to tumor locations or immunological organs to provoke immune cells and induce tumor-specific immune response while regulating immune homeostasis, particularly switching the tumor immunosuppressive microenvironment. This review explores various INP designs and formulations, and their employment in tumor immunotherapy and combination immunotherapy. We also introduce detailed demonstrations of utilizing surface engineering tactics to create multifunctional INPs. The generated INPs demonstrate the abilities of stimulating and enhancing the immune response, specific targeting, and regulating cancer cells, immune cells, and their resident microenvironment, sometimes along with imaging and tracking capabilities, implying their potential in multitasking immunotherapy. Furthermore, we discuss the promises of INP-based combination immunotherapy in tumor treatments.

## Introduction

Cancer is the primary cause of mortality worldwide. Despite the development of several cancer treatment approaches, including surgery, radiotherapy, and chemotherapy, these treatments have shown unsatisfactory therapeutic results. Each therapeutic modality has limitations. Surgery is generally restricted to a limited surgical area, and the effectiveness of radiotherapy and chemotherapy is often compromised owing to difficulties in accurate and efficient targeting, leading to undesirable effects such as tissue injury, drug resistance, and systemic toxicity [1–4]. To overcome these difficulties, extensive research on developing other cancer treatment techniques is in progress, and immune system-reliant cancer immunotherapy has become a huge success in the last decade. The highlighted advantage of immunotherapy is that it induces the generation of circulating effector and memory cells, which not only eliminate primary cancer but also prominently suppress the progression of distant cancer, and efficiently prevent metastasis and reoccurrence of the same type of cancer [5–9]. Immunotherapy expands the targets beyond cancer tissues to cancer-related immune systems. Anchoring and regulating immune cells, including macrophages, DCs, T cells, and immune organs such as LNs, contributes to establishing an internal environment that combats cancer [10–13]. However, similar to other therapeutic techniques, direct administration of immunotherapeutic agents leads to nonspecific targeting, resulting in severe adverse effects. A high dose causes immune imbalance, while a low dose results in no therapeutic response. To address these limitations, nanomedicines have shown potential and promise in cancer immunotherapy. Nanomedicines are generally composed of functional nanocarriers and immunotherapeutic drugs, exhibiting enhanced therapeutic efficiency and reduced side effects, primarily through ligand-guided active drug delivery and specific stimulus-based drug release. Additionally, encapsulating immunotherapeutic agents in nanocarriers improves drug stability, increases half-life, and increases maximum tolerated dose of some drugs, such as antibodies [14–17]. Among several nanocarriers, inorganic nanoparticles (INPs) are undisputed pioneers because of their physicochemical advantages, particularly their specific optical, electrical, magnetic, and catalytic properties, which not only render them useful for drug delivery but also provide platforms for therapeutic, appended imaging, and diagnostic systems [18–22]. However, recent clinical results suggest that immunotherapy alone is insufficient for complete remission and shows more favorable results when combined with other forms of therapeutics. Immunotherapy is compatible with a wide range of conventional anticancer therapeutic techniques because cancer cell death induced by other strategies can aggravate the anticancer immune response [23–27].

**Fig. 1.**
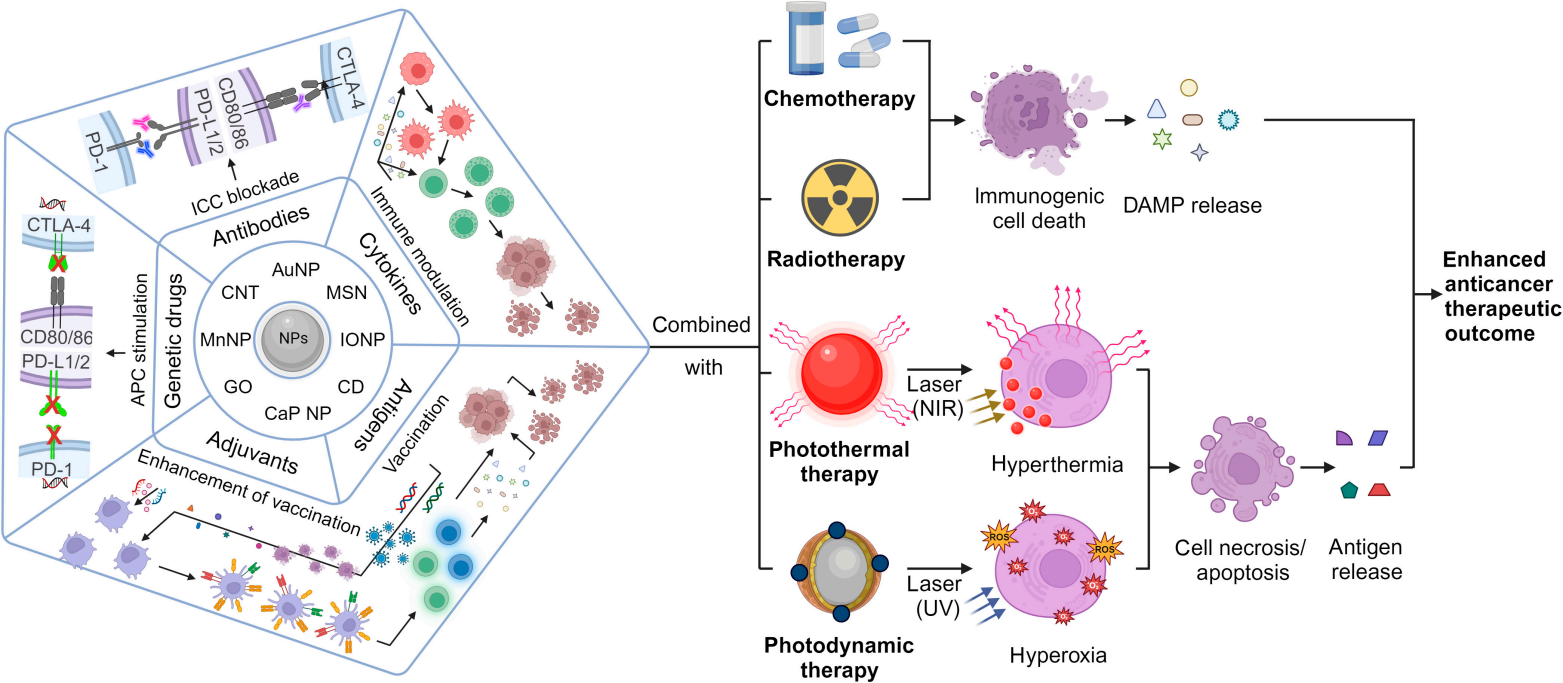
Functionalization of common INPs and their application in combined immunotherapy. A single INP, particularly those with intrinsic properties such as PCE or superparamagnetism, can be modified with immunomodulators and conventional therapeutic agents (chemical drugs, PDT drugs, or radiation agents for synergistic or imaging-guided anticancer therapy with enhanced therapeutic outcomes).

In this review, we provide a concise overview of the engineering concepts and recent advancements in cancer immunotherapy (Fig. 1). Specifically, it focuses on the utilization of INPs as nanocarriers and their combination with immunotherapy. We systematically summarized the most representative INPs, providing a detailed explanation of their alterations and utilization in the field of cancer immunotherapy (Table 1). Furthermore, we enumerated the possible targets of nanoparticulated immunotherapeutic agents and discussed the benefits of employing nanocarrier-based cancer immunotherapy. Finally, we anticipate and consider future advancements in cancer immunotherapy.

**Table 1.** Functionalization of NPs for immunotherapy [22,63,70,76,108,272–291]

Nanocarrier type	Functionalization	Application	Reference
Au nanocarriers	HA and OVA	Vaccination	[272]
	TLR7 ligand 2 and α-mannose	Serve as adjuvant for enhanced vaccination	[273]
	Bcl-2 siRNA	Bcl-2 silencing for enhanced B cell function	[274]
	Imatinib mesylate and anti-STAT3 siRNA	Enhanced inhibition of STAT3 protein for enhanced anticancer effect	[275]
	PD-L1 siRNA	Down-regulated expression of PD-L1 protein for immune checkpoint blockade	[108]
	aPD-1 and aPD-L1	Bind to PD-1 and PD-L1 for immune checkpoint blockade	[276]
MSN	OVA and CpG	DC-targeted delivery and OVA-specific CTL activation for cancer growth suppression	[277]
	OVA and polyIC	Enhanced TAA uptake and DC maturation, and decreased necessary dose of polyIC for cancer immunotherapy	[278]
	GM-CSF, CpG, and OVA	In situ self-assembly of a pocket form MS rods, and recruit, activate, and liberate immune cells (DC) for enhanced immunotherapy	[279]
IONP	MPLA	Immunotherapy and MRI imaging	[280]
Carbon nanomaterials	Formed injectable hydrogel with PEI, OVA mRNA, and R848	Prolonged period of vaccination	[70]
	Alum-Ure B	Served as adjuvant for enhanced immunotherapy	[281]
	Neoantigen, CpG, and aPD-L1	Enhanced and personalized cancer vaccination	[282]
	CpG, aCD40, OVA	Cancer immunotherapy	[63]
	Durvalumab, PEI, and siTrem2	Cancer immunotherapy	[283]
	PDMAEMA and siPD-L1	Cancer immunotherapy	[284]
	B16F10-Ag and CT26-Ag	Cancer immunotherapy	[285]
	OVA-mRNA	Cancer immunotherapy	[286]
	PD-L1 and E3 ligase	Degrade PD-L1, activate STING pathway to promote DC maturation, and reshape the immunosuppressive TME for cancer immunotherapy	[287]
CaP NP	Minicircle DNA encoding BsAbEPH	Fabrication of needle-type CaP nanomaterials for enhanced transfection and antitumor immunotherapy	[288]
	ATP and pOVA vaccine	Enhanced vaccination and promoted costimulatory	[76]
	Zoledronic acid	Improvement of γδT cell proliferation and M1-bias macrophage polarization for enhanced cancer immunotherapy	[289]
	DSPE-PEG2000 and aptamer-DNAzyme	Activated multiple inflammation-related signaling pathways, accelerated DAMP release, and depleted PD-L1 to enhance immunotherapy	[22]
MnNP	Being dopped on ZnO_2_ NP	Together formed NP with ZnO_2_ for synergistic cancer immunotherapy	[290]
	Curcumin and Ca^+^	Activation of cGAS-STING signaling pathway and reprogramming of TME for enhanced cancer immunotherapy	[291]

## INPs for Immunotherapy

### Gold NPs

Gold NPs (AuNPs) have emerged as leading nanocarriers for cancer therapy, in the past decade owing to their advantages from synthesis to functionalization. AuNPs can be easily synthesized with controllable sizes (from several nanometers to micrometers) and shapes (spherical, rod, star, and cubic), and their strong affinity between Au/sulfur and Au/amine endows AuNPs with surface-modifiable capacity. Upon functionalization and conjugation of molecules of different types and functions, such as polymers, biomolecules, and therapeutics, with accordance to the application, AuNPs can be further stabilized and rendered specific properties of targeting, theranostics, imaging, and drug delivery. Owing to the unique localized surface plasmon resonance (LSPR) property of AuNPs, which have strong light scattering and optical absorption, AuNPs are considered novel and efficient contrast agents for computed tomography (CT) imaging.

**Fig. 2.**
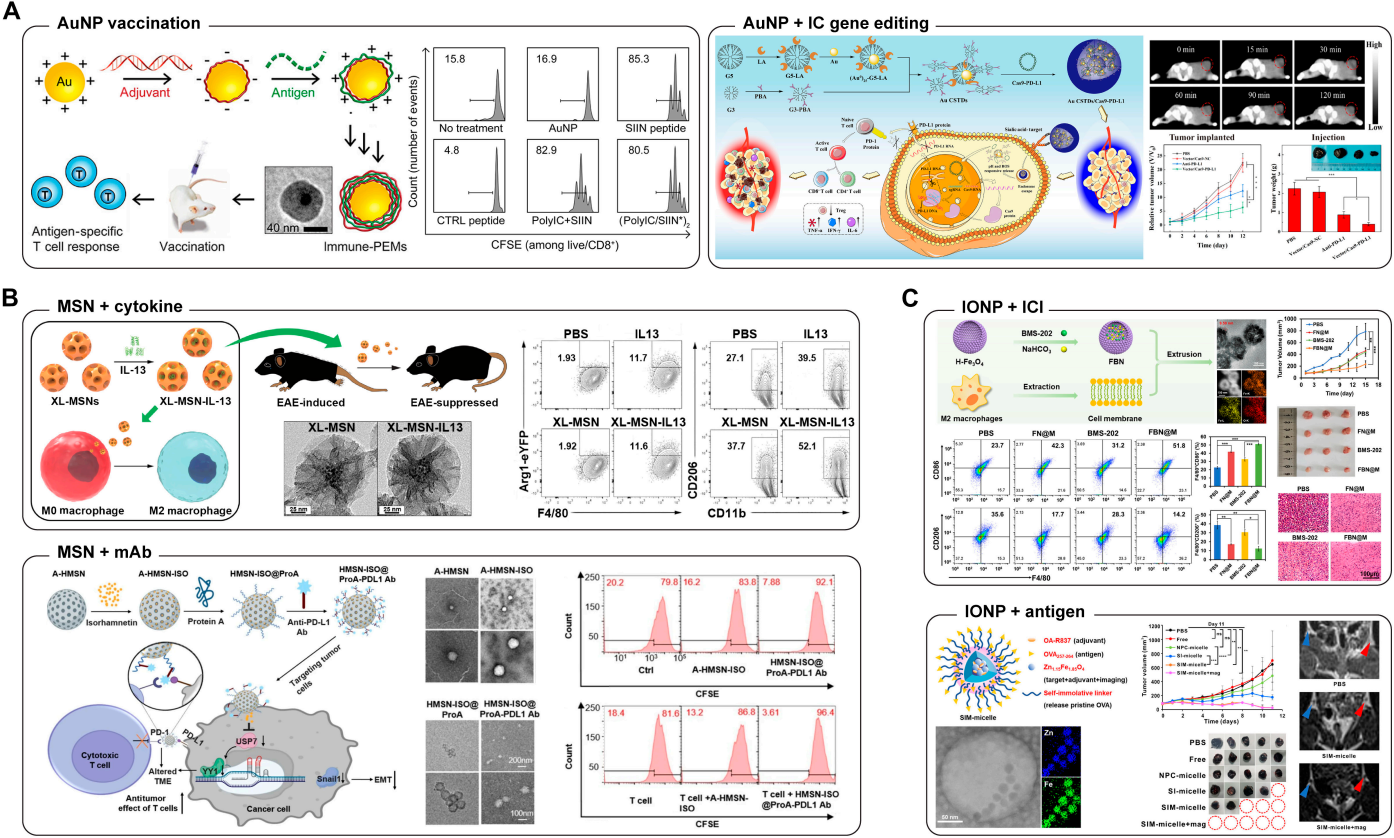
(A) Au nanocarriers carrying therapeutics for tumor vaccination and immunotherapy. Multilayer antigen and adjuvant self-assembly-coated AuNP as a vaccine to promote antigen-specific T cell response. Reproduced with permission from [38]. An AuNP-encapsulating, ROS and pH dual-responsive carrier were complexed with Cas9-PD-L1 for permanent disruption of the PD-L1 gene in cancer cells to facilitate the anticancer immunotherapy. Reproduced with permission from [263]. (B) MSN encapsulating biomolecules for immunotherapy. IL-13-loaded extra-large-pore MSN directs alternative macrophage activation for autoimmune disease treatment. Reproduced with permission from [42]. Anticancer chemotherapeutic agent and anti-PD-L1 antibody dual-encapsulated MSN for TME modulation and tumor progression inhibition. Reproduced with permission from [264]. (C) IONP loading biomolecules for immunotherapy and imaging. A small-molecule PD-1/PD-L1 inhibitor- and gas-forming NaCO_3_-loaded IONP with macrophage membrane wrapping showed pH-responsive gas production-induced drug release, which assists M2 reprograming and PD-1/PD-L1 pathway blockade for enhanced anticancer immunotherapy. Reproduced with permission from [265]. A magnetic metal micelle composed of a metal oxide Zn_1.15_Fe_1.85_O_4_/adjuvant core and an antigen-modified shell were fabricated for visualized LN trafficking and showed efficient CD8^+^ T cell provocation for promoted anticancer immunotherapy. Reproduced with permission from [266].

With increasing attention to immunotherapy in recent years, AuNPs have been predominantly used as vesicles to transport immunotherapeutic cargos (Fig. 2A). Although AuNPs are regarded as bioinert materials, many studies have shown that they can trigger an array of immune responses, alternating the anticancer immunotherapeutic effect. Specifically, one research suggested that AuNPs interacted with extracellular interleukin-1β (IL-1β) to block the production of inflammatory cytokines secreted by the human myeloid leukemia cell THP-1 [28]; another study demonstrated that AuNPs might impair toll-like receptor 9 (TLR9) signaling in macrophages via binding with high-mobility group box 1 (HMGB-1), a nucleic acid (NA) sensor that constitutively associate with TLR9 for activation [29]. The effect of AuNPs on immunomodulation is size and shape dependent. Smaller AuNPs (~5 nm) had a greater influence on regulating immune responses than larger AuNPs (~35 nm) [29,30]. In addition, compared to spherical and cubic AuNPs, rods could be endocytosed by antigen-presenting cells (APCs) involving macrophages and DCs more efficiently and showed stronger response to the secretion of the inflammasome-related cytokines IL-1β and IL-18. In contrast, larger spherical and cubic NPs stimulated increased production of pro-inflammatory cytokines, such as NF-R, IL-6, IL-12, and granulocyte-macrophage colony-stimulating factor (GM-CSF) [31]. The regulatory function of AuNPs in immune responses is predicted by their interaction with critical molecules and/or internalization by particular cells, which either strengthens or weakens the immune response. The behavior of AuNPs is significantly influenced by their shape, surface area, and aspect ratio. Therefore, AuNPs must be meticulously engineered and modified to maximize their application. Although AuNPs have been proven to be capable of modulating immune signaling, they cannot induce strong immune responses to combat tumors. To realize effective cancer suppression and elimination, AuNPs should be further decorated to amplify anticancer signals and/or alter the cancer microenvironment from immune evasive to immune active. The anticancer decoration strategies can be classified into 3 major categories: (a) decoration with cancer vaccines, including encapsulation of specific tumor-associated antigens (TAAs) and adjuvants, which boost APC activation and TAA presentation, promoting tumor-specific anticancer immune responses [32,33]; (b) decoration with genetic therapeutics, predominantly small NA fragments such as small interfering RNA (siRNA), to silence genes that induce immune suppression [34,35]; (c) decoration with antibodies, usually monoclonal antibodies (mAbs) that directly target molecules assisting cancer immune escape [36,37]. Polyinosinic-polycytidylic acid (polyIC) and cytosine-guanine (CpG) motif-containing oligodeoxynucleotides (ODNs) are 2 universal TLR agonists that have been extensively applied as adjuvants with antigens to boost immune responses. Spherical AuNPs with cationic surfaces were alternately layered with an anionic polyIC adjuvant and cationic SIINFEKL peptide antigen through stepwise electrostatic interactions to create polyelectrolyte multilayer (PEM) coatings. Upon intradermal injection, these immune PEMs (iPEMs) were trafficked to tumor-draining LNs and internalized by localized primary DCs, resulting in TLR signaling and antigen presentation. Subsequently, antigen presentation induced the proliferation of antigen-specific cytotoxic T lymphocytes (CTLs) and secretion of more specific effector cytokines, thereby accomplishing efficient vaccination [38]. Therapeutic techniques employing genetic drugs and antibodies share similar strategies, primarily targeting immune checkpoints, such as programmed cell death protein 1 (PD-1)/programmed cell death ligand 1 (PD-L1) and cytotoxic T-lymphocyte associated protein 4 (CTLA-4), to limit their exposure by either down-regulating gene expression or direct binding [37,39,40].

### Silica NPs

The most well-known property of silica NPs, distinguishing them from other INPs, is their mesoporous structure. Mesoporous silica NPs (MSNs) have been designed with various surface decorations for loading and stimuli-responsive drug release, which prevents sensitive drugs from deactivation and digestion and achieves high targeting efficiency [41]. For example, MSNs loaded with IL-13 in their pores showed direct macrophage activation and IL-13 protection [42]. In addition, the mesoporous structure increases the surface area-to-volume ratio, resulting in a higher drug-loading efficiency [43]. Recently, due to the increasing interest in anticancer immunotherapy, MSNs have become potential candidates for trafficking immunotherapeutic payloads to targeted cells and tissues (Fig. 2B).

Similar to AuNPs, MSN-loaded immunotherapeutic cargos are primarily TAAs, genetic therapeutics, and mAb inhibitors [9,44,45]. MSNs demonstrated strong self-adjuvanticity when loaded with TAA for anticancer immunotherapy. A model antigen, ovalbumin (OVA)-decorated MSN, induced only a slightly weaker immune response with an approximately 25-fold lower antigen payload compared with a mixture of OVA and conventional QuilA adjuvant, suggesting that MSN itself is an excellent adjuvant. Intriguingly, the shape of MSNs strongly affects their adjuvanticity, and asymmetric rod-shaped MSN could induce higher expression of CD40 and CD86 maturation markers on APCs than that of spherical MSN, which can be ascribed to that rods were more efficiently internalized by APCs than spherical particles, similar to the findings of rod and spherical AuNPs [46]. Anticancer immune responses can be further enhanced by the co-delivery of antigens and adjuvants, such as OVA and CpG, using MSNs. In addition to the particle morphology, the pore size of MSN has also been reported to stimulate the immune response. MSNs of 80 nm in diameter fabricated for LN targeting were designed with small (7.8 nm, MSNs-S), medium (10.3 nm, MSNs-M), and large pores (12.9 nm, MSNs-L). MSNs with different pore sizes exhibited similar LN-targeting efficiency with significantly different immune activation. MSN-L induced the largest CD4^+^ and CD8^+^ T cell populations in LNs, along with the highest suppression of cancer growth as well as the highest survival rate in animals. This might be due to faster degradation of MSN-L and subsequent extensive antigen exposure to APCs located in the targeted LN [47]. Immune checkpoint mAbs and siRNAs account for a large number of antigens and genetic drugs used in immunotherapy. The mAbs aPD-1 and aPD-L1 inhibit immunosuppressive signals during the immune response by blocking the PD-1/PD-L1 axis. siRNA silencing of PD-1 reduced the expression of PD-1 at the source to stimulate immunosuppression induced by PD-1/PD-L1 interaction [48].

### Iron oxide NPs

Iron oxide (IO) NPs are superior to other INPs because of their intrinsic superparamagnetic nature. IONPs can achieve guided targeting with predictable efficiency using an external magnet. As excellent magnetic resonance imaging (MRI) contrast agents, they enable high-contrast visualization in vitro and in vivo using MRI [49,50]. Therefore, IONPs have recently gained attention for imaging-guided immunotherapy (Fig. 2C). To this goal, IONPs should be modified with functional molecules, usually polymers with functional groups, to arrest therapeutic agents. The antitumorigenic cytokine interferon-γ (IFN-γ) was adsorbed onto dimercaptosuccinic acid (DMSA)-coated magnetic NPs (MNPs) for anticancer therapy. With external magnet application, IFN-γ-adsorbed MNPs could be delivered more specifically to the tumor site, limiting the off-target-induced cytotoxicity in healthy tissues boosting stronger immunogenicity of cancer cells, and inhibiting cancer angiogenesis [51]. Similarly, IONPs have been used to transport therapeutic proteins and NAs to immune organs and tissues. A multifunctional core–shell NP composed of a superparamagnetic Fe_3_O_4_ core and a photonic ZnO shell was designed to deliver carcinoembryonic antigen (CEA) to DCs while functioning as an imaging agent. The Fe_3_O_4_-ZnO NPs were internalized by DCs within a short period, and the NP-pulsed DC-immunized mice exhibited enhanced tumor antigen-specific T cell responses, retarded tumor progression, and higher survival rate. In addition, because of high MRI contrast of NPs, DC migration can be tracked during in vivo administration of NP-laden DCs [52]. Although IONPs are excellent materials for drug delivery and MRI imaging, their size should be considered during particle design to maximize T_2_ relaxivity, drug payload, and cellular uptake. Polyvinylpyrrolidone-coated IONPs (PVP-IOs) with average size of 7.6 to 65.3 nm for IONP cores were fabricated for MRI imaging and macrophage internalization evaluations. In vitro T_2_-weighted MRI images demonstrated that PVP-IOs with the maximum core size displayed the strongest contrast in aqueous solution compared with smaller PVP-IOs of the same molarity. However, in RAW264.7 macrophage cell lines, PVP-IOs with the medium core size of 36.8 nm exhibited the highest cellular uptake. Interestingly, the strongest MRI contrast of the liver was observed in mice that were intravenously administered PVP-IOs with a core size of 36.8 nm and an overall size of 100 nm [53]. Surface modification of IONP alters cellular internalization as well. IONPs with a core size of 12 to 14 nm and a surface coating of DMSA showed the highest T_2_ relaxivity and cell labeling efficiency when they were internalized by cells using the centrifugation-mediated internalization method compared with IONPs of larger or smaller core size with the same functionalization or IONPs with same-sized core but surface decoration with other biomolecules, such as dextran, diethylaminoethyl-dextran, carboxymethyl-dextran, and (3-aminopropyl) triethoxysilane [54]. In addition, relatively small IONPs not only enhance MRI imaging and cellular uptake but also provide larger surface for drug loading, which expedites disease treatment progress [55].

### Carbon nanomaterials

**Fig. 3.**
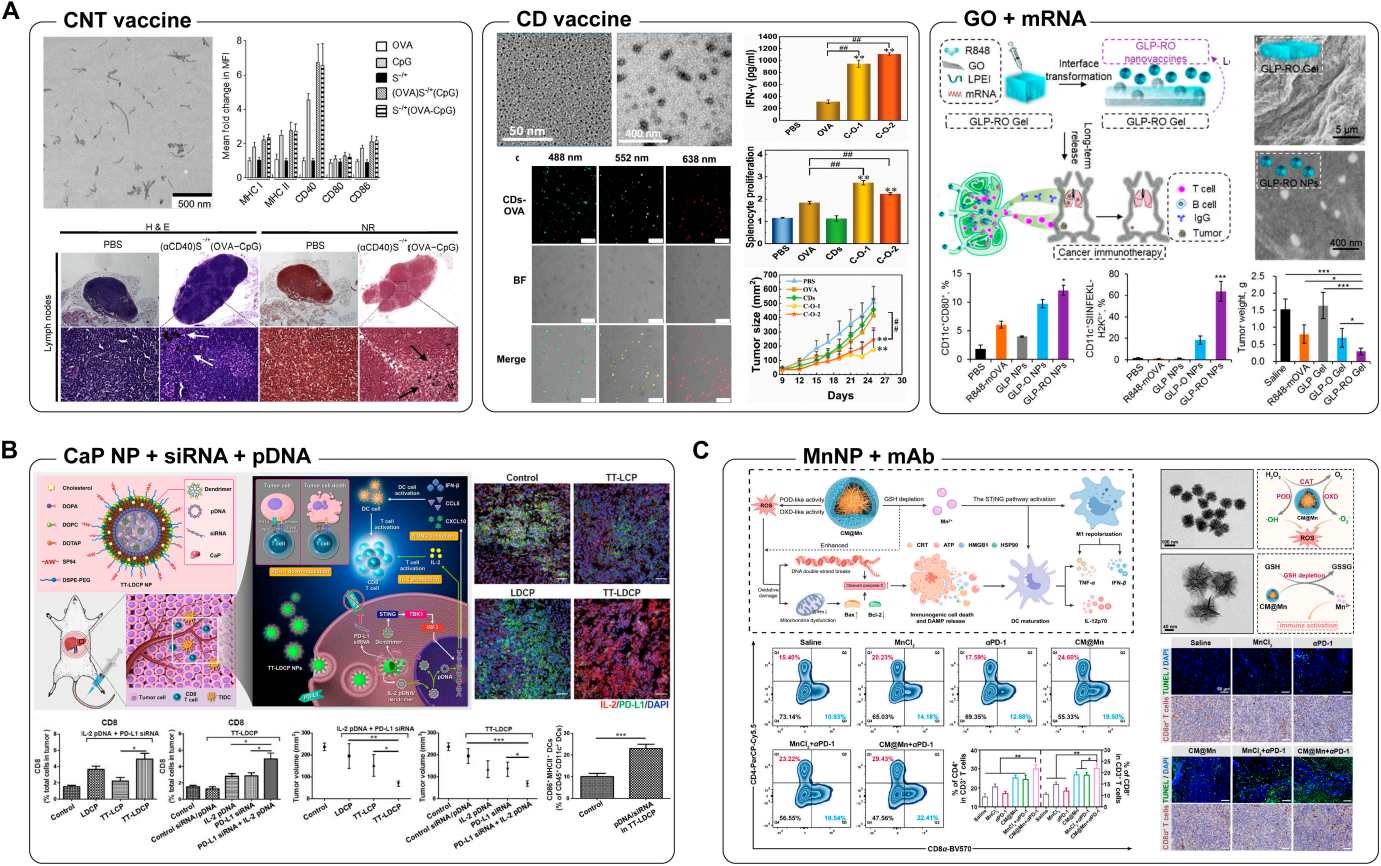
(A) Carbon-based nanomaterials delivering biomolecules for tumor vaccination and immunotherapy. CpG, aCD40, and OVA-loaded MWCNT demonstrate significantly improved OVA-specific immune response and efficient eradication of both orthotopic and pseudo-metastatic tumors. Reproduced with permission from [63]. mOVA- and R848-loaded GO-bPEI injectable hydrogel shows sustained release of nanovaccine, efficient antigen-specific DC activation, and significant tumor regression. Reproduced with permission from [70]. OVA-functionalized CD self-adjuvants with the modification of red-, yellow-, and green-colored luminescent for efficient DC maturation and tumor elimination possess potential for bioimaging and biosensing. Reproduced with permission from [71]. (B) CaP NP together loaded with genetic therapeutics for promoted drug release and immunotherapy. A sophisticated nanocarrier composed of CaP, IL-2 pDNA/PD-L1 siRNA, and thymine-capped PAMAM dendrimer was exploited for pH-responsive drug liberation. The complex showed promises in increased infiltration and activation of CD8^+^ T cells as well as ICB-promoted anticancer immunotherapy. Reproduced with permission from [267]. (C) MnNP with surface functionalization and mAb loading for immunotherapy. Engineered cancer cell membrane-wrapped manganese oxide (CM@Mn) nanoenzyme for TME targeting. CM@Mn induces hypoxia and releases Mn^2+^ in acidic TME, thereby stimulating ICD. Synergism with aPD-1 triggers strong systemic anticancer immunity suppressing distant tumor progression. Reproduced with permission from [268].

Carbon nanomaterials (CNMs) are a remarkable type of drug delivery vehicles and have been employed in immunotherapy. Unlike other inorganic nanomaterials such as AuNPs, which lack degradability and cause bioaccumulation, CNM are considered biocompatible after suitable functionalization and is easily degraded or eliminated by the human body [56–58]. CNM can be classified into diverse types according to their structures; carbon nanotubes (CNTs), including single-walled CNT (SWCNT) and multi-walled CNT (MWCNT), have enormous potential for both cancer treatment and theranostics [59,60] (Fig. 3A). Flexible modifiability imparts CNT with the capacity to load various drugs and contrast agents, and the unique structure endows CNT with the inherent property of strong absorption in the near-infrared (NIR) region, making CNT an outstanding material for photothermal therapy (PTT) [61,62]. Similar to the majority of nanomaterials, CNT may enhance the effects of common adjuvants used in cancer immunotherapy, such as CpG. Regardless of the co-delivery of TAA, both MWCNT and SWCNT conjugated with CpG induced more potent immune responses in vivo [63,64]. Co-administration of CpG and TAA via CNT stimulated TAA-specific immune responses. Co-delivery of CpG, OVA, and CD40 significantly retarded the proliferation of OVA-expressing B16F10 cells in melanoma-bearing mouse models [63]. Not only well-dispersed CNT is a suitable drug carrier, but also some interesting studies have shown that bundled SWCNT can improve protein adsorption; T cell-stimulating antibodies or cytokines were absorbed by SWCNT bundles to evoke immune responses against tumors [65,66].

In addition to CNT, graphene oxide (GO) and carbon dots (CDs) are other CNM forms of interest in the field of immunotherapy. GO is advantageous because of its 2-dimensional (2D) structure, and its wide surface area and abundant hydroxyl and carboxyl groups enable easy functionalization via chemical conjugation. The high hydroxyl and carboxyl group content also contributes to the electronegativity of GO, indicating that GO is suitable for physical modification via electrostatic interactions [67,68]. In addition, GO can sequester molecules through π–π stacking [69]. GO has been electrostatically decorated with polyethylenimine (PEI) to arrest functional DNA or RNA as nanovaccine or gene therapy for immunotherapy. Messenger RNA (mRNA) encoding OVA along with the TLR7/8 agonist resiquimod (R848) was encapsulated in an injectable hydrogel formed by electrostatic interactions between low-molecular-weight PEI and GO. The hydrogel became unstable and gradually transformed into NPs upon liquid solution embedding, such as subcutaneous injection, which achieved a sustained release of mRNA- and adjuvant-containing NPs into the surrounding environment. The results validated that NP release was sustainable for 30 days when the hydrogel was administered in vivo, and the released NPs targeted LN and induced an immune response that efficiently inhibited tumor growth [70]. Based on the same strategy, GO was modified with PEI to electrostatically interact with OVA and CpG, and further layered with polyethylene glycol (PEG) to improve biocompatibility. This formulation along with NLG919, a indoleamine-2,3-dioxygenase inhibitor, was used to treat B16-OVA-melanoma tumor-bearing mice and showed synergistic inhibition of tumor growth by activation of immune system and regulation of tumor microenvironment (TME) [19]. CDs are known as the rising star of carbon-based nanomaterials, which have attracted considerable attention in recent years owing to their versatile properties, including ease of synthesis, manipulation, functionalization, and unique optical and electrical characterizations. The size of CDs is highly tunable from 0.5 to 10 nm per different precursors and synthetic approaches, which ultimately impact the inherent fluorescence of CDs. For immunotherapy applications, CDs have been employed as an adjuvant to interact with TAA via electrostatic interactions to form relatively large nanocomposites (CD-OVA) with a size of approximately 50 nm, as measured by transmission electron microscopy. The CD-OVA efficiently stimulated the expression of the costimulatory molecules CD80 and CD86 on DCs and enhanced the secretion of tumor necrosis factor-α (TNF-α). Upon in vivo application, CD-OVA serves as a vaccine that promotes cellular uptake and strengthens OVA process, resulting in potent inhibition of B16-OVA melanoma growth in C57BL/6 mice by inducing antigen-specific immune responses [71]. CDs have also been functionalized with amphiphilic molecules (ACDs) for RNA delivery. ACDs of 100 to 200 nm in hydrodynamic diameter and approximately 50 mV showed promise for gene delivery. ACDs condensed with green fluorescent protein (GFP)-mRNA showed excellent transfection efficiency within 6 h in different types of cell lines in vitro, and those condensed with OVA mRNA demonstrated significant anticancer efficiency and tumor recurrence prevention when intravenously administered to OVA melanoma-bearing mice.

### Calcium phosphate NPs

Sharing similar compositions with natural bones and teeth, calcium phosphate (CaP) NPs have the potential to serve as biocompatible and biodegradable vehicles for the delivery of anticancer therapeutics. In particular, because of the inherent pH-dependent solubility of CaP NPs, they are considered promising for accelerated and accumulated release of bioactive drugs in acidic environments such as the TME or more acidic endolysosomes upon cell endocytosis [72] (Fig. 3B). Since not all drugs can target the endolysosomal compartment and some drugs are pH sensitive, CaP NP modification is required to protect them from degradation and promote escape from the endolysosomes to deliver the drug to the active site [73]. In addition to transporting common chemical therapeutics, CaP NPs are also ideal scaffolds for generating cancer nanovaccines. Like many other NPs, CaP NPs also exhibit satisfactory adjuvanticity in vaccination and immunotherapy, owing to their suitable diameter, ranging from 1 to 1,000 nm. Recently, disulfiram (DSF), a conventional drug used to treat alcoholism, was coloaded with cupric ions onto lipid-coated CaP NPs (LCP NPs) for cancer treatment. The LCP NPs degraded after accumulation in the tumor to liberate cupric ions and DSF, and the released cargos formed a cytotoxic metabolic complex that induced immunogenic cell death (ICD) of cancer cells, resulting in switching of the immunosuppressive TME to an immunocompetent state and enhancing systemic immune responses [74]. Another study fabricated manganese-calcium phosphate NPs (Mn/CaRis) and employed them as adjuvants to boost immune responses. The Mn/CaRis showed better performance than that of traditional aluminum adjuvants in augmenting both humoral and cellular immune responses to tumor vaccines, thus improving anticancer immunotherapy outcomes. Moreover, in addition to tumor vaccine, Mn/CaRis exhibited amplification effect on prophylactic vaccines as well [75]. Similarly, adenosine triphosphate (ATP) was functionalized on CaP NP (ACP) as both stabilizer and adjuvant and then applied to OVA to manufacture nanovaccine for cancer treatment. This formulation showed efficient DC activation and T cell priming in cellular test as well as tumor growth inhibition when administered to tumor-bearing mice [76].

### Manganese NPs

Manganese NPs (MnNPs), specifically manganese oxide (MnO_2_) NPs, have emerged in the field of cancer immunotherapy in the last decade. The greatest strength of MnO_2_ NPs is that they can have various oxidation states with coordination numbers up to 7.5, making them strong oxidants capable of mediating several oxidation reactions [77]. Owing to this fundamental oxidative property, MnO_2_ NPs have been shown to trigger the conversion of endogenous hydrogen peroxide (H_2_O_2_) into reactive oxygen species (ROS) under acidic conditions such as the TME [78] (Fig. 3C). Moreover, several oxidation states of MnO_2_ NPs enable easy and thorough functionalization of these particles for specific purposes [79–81]. In addition to their flexible reactivity, MnO_2_- and Mn-doped NPs demonstrated high biocompatibility. In vivo administration of Mn-doped MnFe_2_O_4_ NPs showed no irreversible damage to major organs and were eliminated from the kidneys, spleen, and brain of mice on day 7 after injection [82]. Being a superior nanocarrier, MnNPs have been applied for drug delivery in diverse single and combined tumor therapies, including immunotherapy. Moreover, Mn NPs itself have an effect on immune activation. Mn NPs could serve as an oxygen supplier to reduce the expression of the pivotal immune checkpoint PD-L1 in cancer cells by overcoming the hypoxic environment at the tumor site, contributing to the reprogramming of the immunosuppressive TME [4]. Mn NPs were also validated to comprehensively stimulate the cyclic GMP-AMP synthase (cGAS)/stimulator of interferon genes (STING) pathway, a vital signaling pathway related to the activation of the innate immune response combating tumors through the secretion of IFN-I and pro-inflammatory cytokines and by releasing Mn^2+^ ions in the cytoplasm [83]. Similar to AuNPs, Mn NPs can also be employed as adjuvants and have displayed significantly stronger adjuvanticity than that of conventional alum adjuvants for the treatment of live coronavirus and pseudovirus infections in vitro [84]. In addition, because of their strong absorption coefficient and relaxivity, Mn NPs and Mn-doped NPs are suitable contrast agents for both CT and MRI, indicating that these NPs can be used for diagnosis and guided therapy [85,86].

## Targeting

Immunotherapy targets vary greatly from biomolecules to immune organs, and each target has its own merits and drawbacks. In addition, the dosage should also be carefully considered, as a low dose is therapeutically ineffective, whereas a high dose causes severe side effects by systemic immune homeostasis imbalance, which is sometimes fatal.

### Biomacromolecules

#### PD-1/PD-L1 axis

Programmed cell death protein-1 (PD-1) and its ligands PD-L1 and PD-L2 are the most common representative axes of immune checkpoints and have been used as immunotherapy targets for decades. PD-1 is mainly expressed in immune cells, including activated tumor-specific T cells, B cells, DCs, macrophages, natural killer cells, and monocytes, upon chronic antigen exposure [87–89]. PD-L1 is mainly expressed in cancer cells and some APCs and acts as a protumorigenic factor in cancer cells [90]. One major cause of tumor immune escape is the immunosuppressive TME, mainly attributed to the crosstalk between PD-1 and PD-1 ligands through inhibition of the activation, proliferation, and cytotoxic function of CTL [91]. As such, immune checkpoint blockade (ICB) therapy using immune checkpoint inhibitors (ICIs) to block the PD-1 and PD-L1 axes and interrupt their interaction for cancer immunotherapy has been developed as a new milestone, and some clinical trials have achieved promising results [1,92,93]. However, owing to individual differences, high off-target rates, elevated PD-1/PD-L1 inhibitor resistance, and immune-related adverse events (irAEs) caused by the disruption of immune homeostasis upon long-term medication, the universal application of PD-1/PD-L1 blockade drugs is far to go [94–101]. One method to ameliorate these undesired side effects is to introduce nanocarriers to encapsulate the inhibitors. Related research results have proven that the targeting efficiency and immune response can be significantly enhanced by nanocarrier delivery systems, along with reduced off-target rates and side effects [102–106]. For instance, an ingenious AuNP platform with amphiphilic organic ligand protection was synthesized to carry a large payload with less off-targeting uptake, which was demonstrated 40-fold higher cell uptake in the targeted CD8^+^ T cells compared to nontargeted cells [105]. Another example proposed a remotely controlled upconversion NP-based immunodevice that allowed and constrained the generation of effective immune response only within the tumor site without disturbing systemic immunity [106]. Inspired by the intelligent nanotherapeutic devices, nanocarriers have been used to load mAb therapeutics that can directly block the immune checkpoints. An MSNP was employed as a multifunctional platform that co-delivered a PLK1 inhibitor (volasertib) and an anti-PD-L1 antibody for synergistic anticancer therapy by targeting both PLK1 kinase and PD-L1 immune checkpoint. The inhibition of PLK1 kinase selectively kills cancer cells by affecting their mitosis, leading to the up-regulation of PD-L1 expression in surviving cancer cells for a more effective PD-L1 antibody attack [107]. As a result of synergy, the effective doses of both volasertib and PD-L1 antibody were significantly reduced. In addition to facilitating ICB therapy toward the patients that efficiently respond to PD-L1 ICIs, the multifunctional MSNP vehicle might also be potential in promoting the response of patients that possess insufficient PD-L1 to PD-L1 antibodies by up-regulating the PD-L1 expression level. Other metal-based NP or microparticles, such as IONPs, metal–organic frameworks (MOFs), and AuNPs, are also prevalent nanocarrier choices for delivering antibodies, RNAs, or both for PD-1/PD-L1 axis targeting [108–114]. In addition to directly targeting the PD-1/PD-L1 axis, TLR agonists can also be loaded together to amplify the anticancer immunity by eliciting both innate and adaptive immune responses and regulating the TME [16]. Some nanocarriers with specific optical or magnetic properties are capable of multitasking, such as immunotherapeutic drug delivery and imaging or dual therapy involving immunotherapy and PTT [14,115].

#### Cytotoxic T lymphocyte-associated protein 4

Cytotoxic T lymphocyte-associated protein 4 (CTLA-4) is another important immune checkpoint that has recently emerged as a target for cancer immunotherapy. It is a glycoprotein homologous to the CD28 costimulatory molecule and belongs to the immunoglobulin superfamily; it is expressed primarily by T cells and translocated to the cell surface for T cell receptor engagement upon T cell activation [8,116]. As a pivotal biomolecule that regulates T cell homeostasis and self-tolerance, CTLA-4 competes with CD28 to bind to the B7 family molecules, particularly CD80 and CD86 expressed on APCs, resulting in T cell anergy and a compromised anticancer immune response [117,118]. Since CTLA-4 has a superior binding affinity toward B7 compared with CD28, blocking CTLA-4 is considered a promising approach to restore T cell activity [119,120]. Nonetheless, ICI-mediated tumor therapy targeting CTLA-4 is infrequently applied in clinical setting because it shows delayed effects and is sometimes associated with severe side effects, including hepatitis, colitis, and thyroiditis, owing to overactive immune responses [94,121]. Similar to therapeutic agents targeting PD-1 and PD-L1, their effects on CTLA-4 have been developed as nanotherapeutics by conjugating the effective drugs to nanocarriers, particularly those with thermal conversion properties, because hyperthermia-mediated tumor ablation induces immunogenic cell death [26]. SWCNT showed a good capacity for photothermal conversion and were therefore modified with an anti-CTLA-4 antibody (aCTLA-4) for the combined PTT and ICB therapy. In addition to direct thermally induced tumor elimination, tumor cell death leads to elevated TAA release, resulting in the maturation and activation of DCs. In this case, conjugating aCTLA-4 further facilitated CD4^+^ and CD8^+^ T cell activation and infiltration at the tumor sites as well as decreased T regulatory cell (T_reg_) activity [122]. IONPs are also used for hyperthermia therapy because of its magnetocaloric properties [123]. By integrating localized magnetic hyperthermia (MHT) therapy with systemic ICB therapy using aCTLA-4, a more comprehensive anticancer effect could be achieved, with not only tumor regression but also robust inhibition of tumor metastasis and recurrence [124].

However, as previously indicated, the use of ICB treatment is currently limited due to its significant toxicity caused by the disruption of immune homeostasis. Furthermore, owing to inherent variability across individuals, the dosages administered to different patients exhibited significant variation. Hence, the development of a widely standardized ICB therapy and controlled ICI release approach has immense value and significance.

### Cells

Targeting tumor-associated cells is an attractive option for effective immunotherapy. These cells are typically closely linked to the immune system, such as APCs and T cells, or are strongly connected to cancer cell or microenvironment, such as cancer-associated fibroblast (CAF).

#### Dendritic cells

DCs are prominent targets for immunotherapy owing to their crucial role as APCs in initiating a strong immune response mediated by CTL specific to tumor antigens. Briefly, DCs acquire and process tumor-specific antigens. They present the resultant peptide, together with major histocompatibility complex class I (MHC-I) or class II (MHC-II), on the surface of CD4^+^ or CD8^+^ T cells. Furthermore, mature and activated DCs not only display MHC–antigen complex but also exhibit increased expression of CD80 and CD86. This enhanced expression allows effective communication with T cells, facilitating the transmission of costimulatory signals [25,125,126]. In addition, T cell differentiation requires the presence of certain cytokines, including IL-2, to guide the process. Collectively, these processes stimulate specific immune responses against cancer [127]. Conventional DC-based immunotherapy involves the isolation of DCs from individuals, their maturation and activation in vitro using tumor antigens, and reintroduction into patients through transfusion [128,129]. However, these processes are laborious and challenging to implement. The groundbreaking DC-mediated immunotherapy, specifically DC vaccine, involves the presentation of tumor-specific antigens onto nanodevices, which are then directly administered in vivo to initiate a tumor-specific immune response. In some cases, adjuvants are also loaded to augment the immune response. The strategy of loading TAAs and/or adjuvants, usually CpG ODN, into INPs targeting and inducing DC activation for cancer immunotherapy has been extensively discussed in the previous section. Therefore, there is no need to further explore this topic in this context [5,130]. Silencing YTHDF1 protein in DCs is another efficient method, besides CpG stimulation, to increase the expression levels of CD80 and CD86 [12,131]. A nanovaccine platform with AuNP core and dendrimer coating was modified with mannose for DC targeting and compressed with siYTHDF1 to elevate the expression of costimulatory factors. Co-administration of a PD-L1 antibody boosted anticancer immune responses in an animal model with a melanoma tumor xenograft [132].

#### T cells

DCs can be considered as intermediate targets for immunotherapy because they are responsible for presenting TAAs to T cells, thereby triggering a specific immune response against tumors. T cells, specifically CTLs, are direct targets because they directly combat cancer cells. A common method for targeting and activating T cells involves the use of NPs that imitate DCs [132–134]. In this particular instance, NPs are typically disguised with a coating made from cancer cell-primed DC membrane. This coating layer contains not only all DC membrane protein information but also processes that are prepared for presentation [135]. In contrast to DCs, DC-mimic NPs are smaller in size. This allows them to crosstalk more precisely and abundantly with T cells, leading to more effective activation and stronger immune responses [136]. In addition, other functional biomolecules including cytokines and NAs, such as IL-2 or siCTLA-4, can be used together for modification of DC membrane on NPs, to promote CTL proliferation or decrease CTL exhaustion. Although organic NPs, particularly poly(lactic-co-glycolic acid)-based NPs, account for the majority of NP cores camouflaged with DC membranes, some INPs, including AuNPs and MSNP, have also been extensively used as platforms for DC membrane packaging for CTL targeting [137].

Another prevalent T cell target is T_reg_, an important T cell subtype, which plays a critical role in maintaining immune homeostasis, preventing autoimmunity, and suppressing allergy. Unlike CTL, which surface-expresses CTLA-4 only at the activation state, T_reg_ consistently expresses CTLA-4 to regulate excessive overreaction of the immune system. However, in patients with cancer, the inherent property of T_reg_ hinders effective immune surveillance against tumors by establishing an immunosuppressive TME together with other cells such as myeloid-derived suppressor cells (MDSCs), tumor-associated macrophages (TAMs), and CAF. Therefore, targeting T_reg_ to switch the TME to immunocompetent state is of great significance. Nanodevices carrying siCTLA-4 or aCTLA-4 for ICB therapy have been widely employed and are not discussed in detail in this section [8,138].

Chimeric antigen receptor (CAR)-T cell therapy is another ground-breaking pillar of T cell-mediated immunotherapy. T cells are genetically modified with CARs that specifically target malignant tumors, particularly B cell malignancy, under the prevailing conditions. Conventional CAR-T cell therapy consists of 3 main steps: T cell separation, laboratory CAR engineering, and CAR-T cell refusion. The entire technique closely resembles conventional DC-based immunotherapy, including intricate manufacturing procedures, and carries a significant risk, hampering its widespread adoption in clinical practice [139]. Thus, similar to the activation of DCs in living organisms by utilizing NPs, genetic modification of T cells to express CARs can be accomplished in vivo through NP delivery. This approach represents a potential strategy for creating universally applicable targets for immunotherapy [140]. Despite the use of biomolecule-based nanovesicles, such as liposomes and exosomes, and polymer-based complexes, inorganic nanomaterials such as quantum dots, CNTs, AuNPs, and MSNPs have also shown significant potential for in vivo gene editing of T cells and have been experimented [141].

#### Macrophages

Macrophages are predominant components of the TME and are involved in tumor development. They can be polarized into 2 basic phenotypes under different stimulations: M1-type macrophage (M1) and M2-type macrophage (M2), which are also referred to as TAM [142,143]. Concisely, M1 can be stimulated by IFN-γ, lipopolysaccharide (LPS), TNF-α, or GM-CSF, which then triggers the activation of TLR signaling pathways [144]. They have beneficial effects on the elimination of infections and malignant cells. M1 macrophages exert their antitumor activity in several ways. They express high levels of antigen-presenting MHC complexes to activate the adaptive immune response to combat tumors [145,146]. They act directly on cancer cells by generating nitric oxide (NO), ROS, and reactive nitrogen species (RNS). Moreover, they promote inflammatory responses by secreting pro-inflammatory cytokines [13,147–149]. In contrast, M2 macrophages are activated by several cytokines. Accompanied by the increased production of polyamines and ornithine through the arginase pathway, they participate in parasite clearance and homeostasis, including tissue remodeling and regeneration, wound healing, and anti-inflammation [150,151]. Two approaches have been developed to regulate M2 macrophages in cancer immunotherapy. One approach involves depleting M2 macrophages to transform the TME into an M1-biased antitumor environment that can prevent tumor growth [152]. Another approach involves exploiting the plasticity of differentiated macrophages to convert M2 into M1 under specific circumstances [153]. Consequently, M2 macrophages can be repolarized to M1 macrophages and prevent tumors. Both approaches were accomplished using nanomodulators, employing either organic or inorganic NPs as platforms [154–156].

#### Cancer-associated fibroblasts

CAFs are another dominant and abundant cell type responsible for constructing and modifying the extracellular matrix (ECM) framework within the TME. Nevertheless, because of the high heterogeneity and deficiency of specific markers, identification of CAF remained ambiguous for long period. Fortunately, owing to recent technological advancements, such as single-cell RNA sequencing, CAF can now be divided into 2 main subtypes: myofibroblastic CAF (myCAF) and inflammatory CAF (iCAF) [157,158]. Although the exact role of CAF subtypes in tumor progression is not yet fully understood, several studies have demonstrated that myCAFs can induce the expression of PD-1 and CTLA-4 in T_reg_ through a positive feedback loop, suggesting that myCAFs hold potential as viable targets for cancer immunotherapy [159,160]. CAFs exhibit higher metabolic activity than that of regular fibroblasts. Although fibroblasts are typically considered inert in normal tissues and can be activated to assist in wound healing and tissue regeneration, CAFs are more metabolically active. They induce the production of various growth factors and pro-inflammatory cytokines to promote angiogenesis and recruit immunosuppressive cells to establish a TME capable of assisting tumor immune evasion [161,162]. As a burgeoning target, CAFs have been tested using various strategies, including ICB therapy by reducing the exposure of PD-1 or CTLA-4, blockade of the critical surface marker CXCL12 and the corresponding receptor CXCR4, depletion of fibroblast activation protein (FAP) accompanied by anticancer vaccination, normalization of CAF to reduce the deposition of ECM that impedes CTL infiltration, inhibition of NOX4 to prevent the activation of CAF, and even inactivation of activated CAF [163,164]. These strategies can be integrated with nanocarriers to enhance their therapeutic effects.

### Sites

#### Tumor-draining lymph node

As an immune cell-resident organ, LN is another eligible drug target in immunotherapy. LN-targeted delivery can be briefly categorized into 2 major types: direct intranodal injection and lymphatic drainage followed by other methods involving systemic, subcutaneous, intradermal, intramuscular, and intraperitoneal injections [6,165–167]. Intranodal delivery, as the name indicates, involves direct injection of drugs or platforms with drug payloads into the LN. The advantage of this approach is that it minimizes off-targeting and concentrates the therapeutics to the LN, which reinforces the exposure of the resident immune cells to the drug and contributes to enhanced interaction between them, and finally resulting in enhanced immune responses [11,168]. For instance, intranodal injection of tumor-specific antigens and adjuvants has been shown to be effective in enhancing anticancer immunity by enhancing their contact with APC and their subsequent processing and presentation [169]. In addition, tumor lysates are alternative agonists for priming tumor-specific immune responses via intranodal injection [170]. Immune cells can also be administered intranodally. In vivo reinfusion of antigen-pulsed DC is a typical immunotherapy approach that has exhibited significant anticancer effects; however, direct intranodal injection showed stronger T cell response induction than that of intravenous DC refusion [171,172]. In addition to activating the immune system, modulation of immune cells is another alternative for enhancing the immune response. Some cytokines can be directly injected into LNs to control the phenotype or function of immune cells. For example, IL-2 is essential for T cell proliferation and has therefore been delivered together with tumor antigens to strengthen adaptive immunity [173]. However, compared with other administration approaches, intranodal delivery is more invasive and less accessible [174]. Moreover, some therapeutics injected intranodally can be rapidly eliminated from LNs, ultimately leading to poor immunological modulation [165].

LN-targeted delivery of injected immunomodulators by direct afferent lymphatic drainage is highly dependent on their physicochemical properties, particularly their size. Although small-sized immunomodulatory agents can be easily drained into the LN, they are also rapidly cleared, and their poor retention time is insufficient to pulse anticancer responses [175–177]. In this case, larger NPs with immunomodulatory functionalization are a better choice. The optimum size of NPs for lymphatic drainage as well as LN retention is a diameter of 10 to 200 nm, and NPs of smaller or larger size exhibit poor LN retention or poor lymphatic drainage, respectively [10,175]. In addition to size, the hydrophobicity and surface charge of NPs also influence their LN-targeting capacity. Hydrophilic NPs with anionic or neutral surfaces are more likely to be drained [178,179]. To further facilitate immunological activation after lymphatic drainage, NPs can be modified with biomolecules, typically antibodies against specific immune cell surface markers, to enhance their interactions with immune cells. NPs modified with anti-CD11c showed enhanced interaction with DC, and those decorated with anti-CD8 facilitated CTL binding [10].

#### Tumor microenvironment

The TME and its role in the development and advancement of various cancers has been acknowledged over the last decade. The TME is a complex system consisting of cells derived from mesenchymal, endothelial, and hematopoietic lineages organized within the ECM. These cells interact closely with cancer cells and play a significant role in tumor development [180,181]. The interaction between the tumor and TME plays a crucial role in influencing the advancement of cancer, by either promoting or inhibiting its progression [182,183]. Although the TME initially has anti-malignant effects in early-stage tumors, many cancer cells can withstand this suppression and subsequently alter the TME to promote the malignant behavior [182,184]. The TME is complicated and diverse during the last stage of solid tumors. First, rapid proliferation of cancer cells induces several irreversible events, which cause metabolic remodeling of cancer cells and an adaptation of the TME to the new setting, and the TME infrastructure is influenced by various immune and nonimmune cell types. The complex TME induces disorganized biological processes, including secretion of various cytokines and chemokines, alteration of metabolites, hypoxia, angiogenesis, ECM remodeling, interstitial pressure, and pH changes [185–188]. These factors contribute to the development of a chronic inflammatory, pro-angiogenic, and immunosuppressive environment in cancer [189,190]. Over the last decade, the TME has been recognized as a favorable setting for the development of new and effective anticancer drugs. The basic TME targets can be categorized into 2 types. One targets TME cells that exert immunosuppressive effect, including tumor-infiltrating T_reg_, M2 macrophages, MDSC, CAF, professional APCs such as DCs, and tumor-combating effector cells, whose functions are inhibited by the immunosuppressive TME, involving CTL [191]. The other factor is the environment itself, including ECM, hypoxia, and acidic pH [192]. TME delivery approaches can be divided into 2 types: systemic and intratumoral. Systemic delivery is realized by intravenous injection, and efficient delivery can be achieved by active targeting that employs tumor-targeting molecules to anchor tumor sites or passive tumor accumulation is mediated by particle size and surface charge [193–195]. Intratumoral delivery is accomplished by direct injection of therapeutic substances inside the tumor. Compared to systemic delivery, intratumoral delivery has always shown better therapeutic effects because the proportion of injected drug for utilization is significantly elevated. However, as not all tumors are physically accessible in the absence of surgery, systemic delivery approach is universally applicable [196–198].

## Combination Therapy

Although immunotherapy has demonstrated advantages in pulsing adaptive immune responses and preventing tumor recurrence, it is not universal for all types of tumors. In addition, the dose differs significantly depending on the individual, and an overdose can cause severe adverse effects [199,200]. To maximize the anticancer effect with reduced adverse effects, higher survival rate, and prolonged survival, combination therapy is occasionally preferred over single immunotherapy. Current therapeutic methods used in combination with immunotherapy include chemotherapy, PTT, photodynamic therapy (PDT), and radiation therapy [201–204]. The common combination therapy and their applications were summarized and listed as Table 2.

**Table 2.** Combination of immunotherapy with other therapeutic strategies [122,269,270,292–304]

Combined therapeutic strategy	Carrier	Functionalization	Application	Reference
Chemotherapy	IONP	EBP, polyIC, DOX,	Enhanced tumor targeting and combined immunotherapy and chemotherapy	[292]
	CD	AEAA, DOX, Fe ion, LOS, and Asp-Ala-Thr-Gly-Pro-Ala peptide crosslinker	Combined immunotherapy with chemotherapy	[293]
	CD	FA, Cu, and aPD-L1	Combined ferroptosis and immunotherapy for tumor eradication	[294]
	MnNP	DOX and phospholipid	Induction of immune cell maturation, activation, and up-regulation of cytokine release for enhanced combined immunotherapy and chemotherapy	[269]
PTT	AuNP	TAA and DC biomolecules	Stepwise intra-cancer cell and DC-generated AuNP for combined immunotherapy and PTT	[270]
	AuNP	CpG	Fabricate of hydrogel using complementary DNA chains for combined immunotherapy and PTT	[295]
	IONP	MIQ and ICG	Combined immunotherapy with interventional PTT for PC treatment, together with MRI guidance and temperature monitoring	[296]
	IONP	Being coated on platelets together with aPD-L1	Combined immunotherapy with PTT for postsurgical prevention of tumor reoccurrence	[297]
	IONP	PDA, PEG, RGD, anisamide, and R848	Combined immunotherapy with PTT for anticancer therapy and MRI for tumor diagnosis	[298]
	GO	PEI, PEG, and CpG	Combined immunotherapy with PTT	[299]
	GO	MTX and SB-431542	Combined immunotherapy with chemotherapy and PTT	[300]
	CD	aCTLA-4	Combined immunotherapy with PTT	[122]
	CD	Al, mannose and CpG, and formed hydrogel with oxidized dextran	Combined immunotherapy with PTT for tumor eradication	[301]
	MnNP	Being coated with mPEG-b-PHEP and IR780 dye	Combined immunotherapy with PTT	[302]
PDT	MSN	MMP-2, aPD-1, Ce6, and PTX	Facilitate tumor accumulation, cellular uptake, immune checkpoint blockade, and combine immunotherapy with chemotherapy and PDT	[303]
Gas therapy	IONP	l-Arg and PAA	Reprogram M2 TAMs toward M1 TAMs, release proinflammatory cytokines, and recruit T cells, and combined gas therapy with immunotherapy for anticancer treatment	[304]

### Chemo-immunotherapy

**Fig. 4.**
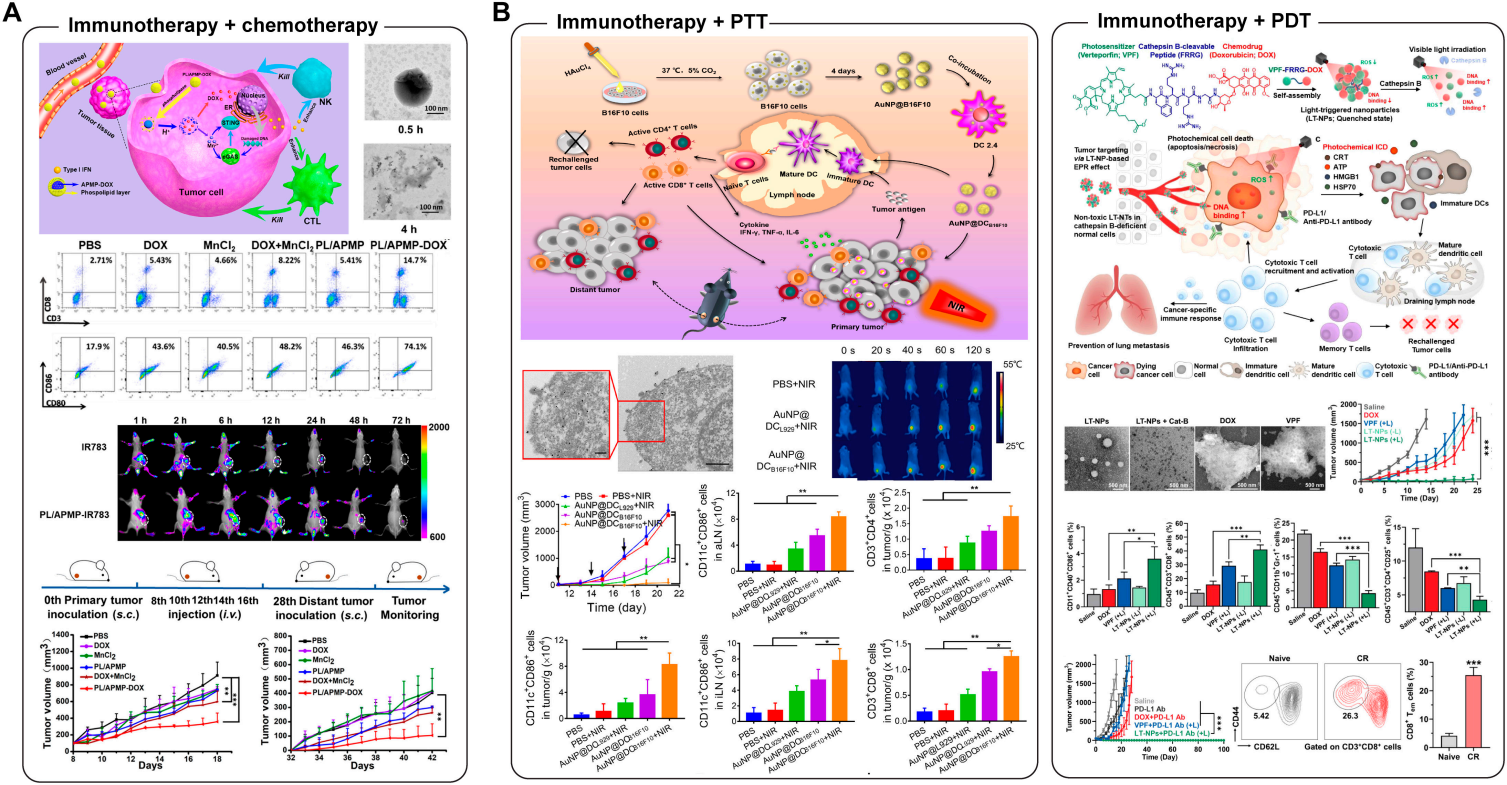
(A) Immunotherapy combined with chemotherapy for tumor treatment. Manganese-based nanoactivator loaded with doxorubicin (DOX) and phospholipid shows prolonged systemic circulation and induces ICD via DOX as well as sensitize cGAS-STING pathway by released Mn^2+^ in cancer cells, thus boosting both innate and adaptive anticancer immune responses and eliminating tumor by combined chemotherapy and immunotherapy. Reproduced with permission from [269]. (B) Immunotherapy combined with phototherapy for tumor treatment. Immunological AuNPs generated stepwise in cancer cells and DCs presenting tumor antigens for combined PTT and immunotherapy, which exhibit enhanced biosafety, rapid hyperthermia induction, and significant evocation of tumor-specific immune response. Reproduced with permission from [270]. Manufacturing of visible light-triggered prodrug NPs with the modification of DOX and PS to enhance ICD and immune response by synergistic chemotherapy and PDT at the tumor site, and combining them with PD-L1 checkpoint blockade-mediated immunotherapy for tumor eradication and cancer recurrence prevention. Reproduced with permission from [271].

Chemo-immunotherapy involves a combination of traditional chemotherapy and emerging immunotherapy. Although some chemotherapeutics can trigger dose-dependent immunosuppression, the combination of chemotherapy and immunotherapy in some cases may maximize the therapeutic efficacy through potential synergistic effect [23,24] (Fig. 4A). One major chemotherapy actively combined with immunotherapy is cytotoxic, because it induces ICD, which can boost the adaptive immune response [205]. The underlying mechanism is that the ICD of cancer cells leads to the release and relocation of damage-associated molecular patterns (DAMPs), which act as strong adjuvants to pulse immune cells and potentiate immunotherapy [206,207]. Typical ICD-associated DAMP progression includes cell surface expression of calreticulin (CRT), ATP discharge, and post-apoptotic release of HMGB1. Exposure to CRT leads to elevated endothelial expression of adhesion molecules, resulting in promotion of lymphocyte infiltration into the tumor tissue and enhanced cancer immunotherapy. Released ATP serves as a “find-me” signal to attract APCs and recruit DCs to migrate to LNs. In contrast, released HMGB1 interacts with TLR4 in DCs to decelerate the degradation of phagocytic antigens and augment their presentation [208–211]. In addition to the above DAMPs, the release of annexin A1, heat shock proteins 70 and 90 (HSP70 and HSP90), TLR3, TLR9, and other cytokines and chemokines also play roles in maturation and recruitment of APCs and T cells [212–214]. ICD is capable of enhancing adjuvanticity, and several studies have implied that it enables stronger antigenicity in cancer cells. Some chemotherapeutic drugs significantly up-regulate antigen expression. For example, gemcitabine and topotecan can induce elevated expression of human leukocyte antigen (HLA) through activation of the nuclear factor κB (NF-κB)/IFN-β/MHC-I signaling axis, which leads to amplification of tumor-specific immune response [215]. In addition to administering chemotherapeutic agents that are capable of inducing enhanced ICD to provoke immune cells, employing functionalized NPs for promoted and precise targeting as well as localized stimuli-responsive drug release also helps with specific immune response and reduced systemic toxicity. Although organic NPs are more prevalent carrier choices for chemo-immunotherapy, INPs still have a place in this field due to their unique properties. For instance, some INPs serve as more than carriers, and they can also be stimuli-susceptible drug precursors; some INP carriers not only deliver drugs but also impart imaging-guided functions [216–220]. An iron-carbonyl complex was designed for chemodynamic immunotherapy since it could be initiated by both glutathione and H_2_O_2_ to generate CO and ferrous ions, which could induce mitochondria damage as well as oxidative stress, resulting in ICD-mediated immune response [217]. Another example showed that prodrugs loaded on nanogapped AuNPs sensitive to pH and glutathione could work together with photoacoustic imaging to help with chemo-immunotherapy and imaging of the deep tumor area in the NIR-II window [216]. In some situations, elaborated INP platforms could be better choices for multi-tasking purposes with more concise fabrication steps in comparison to some organic NPs, making them promising candidates in combined immunotherapy.

### Radio-immunotherapy

Radiotherapy is another therapeutic approach that enhances the adjuvanticity and antigenicity of cancer cells to boost tumor-specific immunity. Therefore, they are suitable for combination with immunotherapy [221]. Similar to cytotoxic chemotherapy, radiotherapy can also induce ICD, followed by the surface translocation of CRT and release of annexin A1, HSP70, HSP90, HMGB1, and other molecules, resulting in enhanced recruitment of APCs and T cells [222–224]. Moreover, radiation down-regulates the expression of the macrophage “Do not eat me” signal, CD47, which collaborates with the elevated “eat me” signal CRT, to further enhance the tumor-specific antigen presentation [225]. In addition to ICD, radiation enhances the presentation of tumor antigens by up-regulating the expression of MHC-I and induces the damage and release of DNA to up-regulate the expression of the type I interferon pathway and boost immune responses via the cGAS/STING pathway [226,227]. In addition to directly damaging DNA, radiation induces ROS generation, which oxidizes DNA and proteins, and increases their antigenicity [228]. However, as radiation indiscriminately attacks cancer and normal cells, it also causes the death of immune cells that infiltrate the TME [229]. It also promotes the infiltration and aggregation of immunosuppressive MDSC and T_reg_ and simultaneously reduces the penetration of CTL into the TME. Together, these circumstances lead to the generation of a more immunosuppressive TME, weakening the effect of immunotherapy and assisting tumor progression [230,231]. Despite being challenging, combining radiotherapy with immunotherapy is prospective to minimize undesirable side effects, and using INPs for image-guided drug delivery could further reduce the toxicity toward normal tissues [3,18,232–234]. Based on this concept, a radiation-responsive snowflake-like Au nanocarriers (S-AuNC) were loaded with aPD-L1 for synergistic radio-immunotherapy. External radiation not only induced ICD for immune evocation but also mediated S-AuNC deformation and aPD-L1 liberation for ICB-mediated immunotherapy. In addition, Au could be a splendid contrast agent for CT imaging, which achieved image-guided radiation, resulting in minimized systemic immune-related adverse effects [233]. Likewise, other INPs such as activation and guiding of irradiation by x-ray (AGuIX) NP were used as radiosensitizers and were together administered with aPD-L1 for synergistic therapy. Compared to conventional radiotherapy, AGuIX NP/aPD-L1-mediated radio-immunotherapy significantly increased the infiltration of effector CD8^+^ T cells and effectively alleviated the immunosuppressive TME [18]. Therefore, NP-mediated combined radio-immunotherapy showed superiority over either radiotherapy or immunotherapy, because its imaging-guided property could realize controlled treatment to maximize the outcome while minimizing the adverse effect.

### Photo-immunotherapy

Phototherapy, which principally involves 2 typical categories, PTT and PDT, is an important cancer treatment modality. PTT and PDT offer significant benefits such as noninvasiveness, low systemic toxicity, and precise tumor targeting [235–237].

PTT entails injecting materials with a high photothermal conversion efficiency (PCE) into the human body, followed by tumor tissue accumulation via passive or active targeting. When materials are exposed to external light sources, such as NIR laser, the light energy is converted into heat, producing local hyperthermia and inducing cancer cell necrosis or apoptosis, leading to tumor elimination [238,239]. One of the most critical factors in inducing significant PTT is the selection of photothermal transduction agent (PTA) with efficient PCE. PTT encompasses different categories of converters, including inorganic photothermal converters such as Au nanomaterials and organic photothermal converters such as polydopamine NPs [240,241]. Compared with inorganic Au nanomaterials, which exhibits a high PCE of NIR, resulting in damage of normal tissues in the tumor surroundings, organic polydopamine NPs exhibit excellent compatibility with living organisms and are an optimal choice for PTT [242]. PTT not only directly ablates tumors but also contributes to ICD, which induces the release of DAMPs and TAAs from dying cancer cells to enhance the immune response. In addition, elevated temperature at the tumor site expedite blood circulation, which promotes the accumulation of systemically administered small molecules such as immunotherapeutic agents inside tumors [26]. Therefore, the combination of PTT and immunotherapy synergistically enhances primary tumor eradication and distant metastasis (Fig. 4B).

In contrast, the PDT regime is highly oxygen dependent. This includes the application of photodynamic molecules known as photosensitizer (PS), followed by the induction of singlet oxygen (^1^O_2_) generation by reacting with O_2_ or ROS via direct oxidation of biomolecules under photo-irradiation [243,244]. However, most conventional PS agents, such as chlorine or phthalocyanine, show poor solubility in water, limiting their in vivo application. To address this deficiency, nanocarriers are typically introduced to load PS agents for targeted trafficking with efficient drug delivery. In this case, PS agents are usually encapsulated in the core area via hydrophobic interaction or shielded by a hydrophilic layer in the intermediate phase of solid NPs [245–247]. The modified NPs were multitasked with cancer elimination through synergistic PDT and immunotherapy by cofunctionalizing with immunotherapeutic agents such as antigens, antibodies, adjuvants, and NAs (Fig. 4B).

## Summary of Current Processes in Cancer Immunotherapy and Perspectives

INPs have been widely employed in conventional chemotherapy and phototherapy because of their biocompatibility, high drug-loading capacity, enhanced targeting efficiency, and unique physicochemical characteristics. However, unlike organic NPs, most of which are designed to degrade spontaneously, many INPs cannot be easily eliminated in vivo, making them arduous for clinical translation due to the potential toxicity. Nevertheless, clinically administering INP-based therapeutic agents is not an impossible gulf. There have been well-designed biodegradable INPs, such as MnO_2_, iron-carbonyl complex, calcium ore NPs, and so forth, as prodrugs or drug carriers for in vivo application. In addition, stimuli-triggered deformable INPs, partially degradable organic/inorganic hybrid NPs, and INPs with a diameter smaller than 5 nm can be easily renal cleared and therefore are promising candidate for clinical translation [4,18,83,84,217,233]. In fact, there have been a few approved INP drugs used for clinical treatment, and more have been successfully progressed to clinical trials [248].

With development in cancer immunotherapy and discovery of self-adjuvanticity of a large number of INPs, the application of INPs as carriers for immunotherapeutic agents, such as mAbs and genetic drugs that block immune checkpoints, has emerged in the last decade [249]. Immunotherapeutic agents regulate immunosuppressive signaling to switch the “cold” (immunosuppressive) TME to the “hot” (immunocompetent) state, ultimately provoking systemic anticancer immune signals. Inorganic hafnium oxide NP-based therapeutics (NBTXR3/Hensify) have been included in many clinical trials, and one of them has been approved for radiotherapy (CE Mark, 2019). Because this platform also triggered enhanced antitumor immune response, the NP has potential for immunotherapy and combined radio-immunotherapy, and its clinical trial for immunotherapy is also under progress (NCT03589339) [7,250,251]. However, several studies have shown that cancer immunotherapy is highly individually biased; some patients respond strongly to immunotherapeutic agents, whereas others have a weak or even no response to the same formulation [94,252]. Therefore, to achieve potent tumor elimination while preventing tumor metastasis and recurrence, combined therapies integrating immunotherapy with other anticancer therapeutic modalities have burgeoned [253–255]. In recent years, advanced clinical therapeutics have been explored for compatibility with immunotherapy for synergistic therapeutic effect, such as the combination of cryosurgery with immunotherapy, which provides a much safer technology and achieves complete tumor elimination, including operative cancers and distal and metastatic cancers of the same type [135,256,257]. In addition to combining surgery and co-delivering immunotherapeutic agents with drugs having specific anticancer effects on the same substance, anticancer outcomes can also be improved by proper nanocarrier selection. Nanocarriers with inherent characteristics, such as the ability to generate heat and produce ROS and singlet oxygen under irradiation, or properties of guided tumor targeting in the presence of an external force, are promising candidates to exert synergistic anticancer effect with modified immunotherapeutic agents [258,259]. Both Au and mesoporous silica nanocarriers possess inherent self-adjuvanticity and have been widely used for antigen delivery [12,260]. Au, IO, and carbon nanocarriers have demonstrated substantial PCE, enabling their use for integrating immunotherapy with PTT [261]. Likewise, Mn NPs can function as O_2_ suppliers and therefore can be used as carriers to enable hyperoxia-enhanced immunotherapy [4].

In addition to conventional immunotherapeutics, cell membranes, such as tumor and immune cell membranes presenting TAAs, have become novel stimuli that evoke specific immunity. Cell membrane-camouflaged nanocarriers contain complete TAA information, facilitating the induction of tumor-specific immunity; in addition, subcutaneous injection of small-sized nanocarriers enables more precise LN targeting and subsequent immune cell priming. Cell membrane-coated nanocarriers can accumulate in tumor tissues via ligand-mediated active targeting and size-dependent passive targeting when administered intravenously and then modulate the TME to stimulate the anticancer immune response. Additionally, augmenting targeting efficiency and reducing off-target effects can be achieved through the modification of nanocarriers with cell membranes engineered with synthetic ligands [135,177]. Nanocarriers with appropriate decoration also facilitate the in vivo generation of functional immune cells, such as CAR-T cells and M1 macrophages [140,262]. Considering the similar physicochemical (small size) and unique biological characteristics of various inorganic NPs, as well as ease of administration, effective targeting, and potential to enhance cancer elimination and prevent cancer recurrence when employed together with other anticancer therapeutic approaches, we anticipate that NPs hold great potential for advancements in anticancer therapy.

## Data Availability

Data availability is not applicable to this article as no new data were created or analyzed in this study.
